# Polygenic scores for autism are associated with reduced neurite density in adults and children from the general population

**DOI:** 10.1038/s41380-025-02927-z

**Published:** 2025-02-24

**Authors:** Yuanjun Gu, Eva Maria-Stauffer, Saashi A. Bedford, APEX consortium, Yuanjun Gu, Deep Adhya, Carrie Allison, Bonnie Ayeung, Rosie Bamford, Richard Bethlehem, Tal Biron-Shental, Graham Burton, Jonathan Davies, Joanna Davis, Dori Floris, Alice Franklin, Lidia Gabis, Daniel Geschwind, David M. Greenberg, Alexandra Havdahl, Alexander Heazell, Rosemary Holt, Matthew Hurles, Yumnah Khan, Meng-Chuan Lai, Madeline Lancaster, Michael Lombardo, Jose Gonzalez Martinez, Jonathan Mill, Mahmoud Koko Musa, Kathy Niakan, Adam Pavlinek, Lucia Dutan Polit, Marcin Radecki, David Rowitch, Laura Sichlinger, Deepak Srivastava, Alexandros Tsompanidis, Florina Uzefovsky, Elizabeth Weir, Xinhe Zhang, Hilary Martin, Simon Baron-Cohen, Varun Warrier, iPSYCH-autism consortium, Anders Borglum, Jonas Bybjerg-Grauholm, David M. Hougaard, Ole Mors, Preben Bo Mortensen, Merete Nordentoft, Thomas Werge, Jakob Grove, Rafael Romero-Garcia, Jakob Grove, Anders D. Børglum, Hilary Martin, Simon Baron-Cohen, Richard A. I. Bethlehem, Varun Warrier

**Affiliations:** 1 Department of Psychiatry, University of Cambridge, Cambridge, CB2 8AH UK; 2 Autism Research Centre, Department of Psychiatry, University of Cambridge, Cambridge, CB2 8AH UK; 3 Department of Medical Physiology and Biophysics, Instituto de Biomedicina de Sevilla (IBiS), HUVR/CSIC/Universidad de Sevilla/CIBERSAM, ISCIII, 41013, Sevilla, 41013 Spain; 4 The Lundbeck Foundation Initiative for Integrative Psychiatric Research, iPSYCH, Aarhus, 8000 Denmark; 5 Center for Genomics and Personalized Medicine (CGPM), Aarhus University, Aarhus, 8000 Denmark; 6 Department of Biomedicine (Human Genetics) and iSEQ Center, Aarhus University, Aarhus, 8000 Denmark; 7 Bioinformatics Research Centre, Aarhus University, Aarhus, 8000 Denmark; 8 Human Genetics Programme, Wellcome Sanger Institute, Wellcome Genome Campus, Hinxton, CB10 1SA UK; 9 Department of Psychology, University of Cambridge, Cambridge, CB2 3EB UK; 10 Wellcome-MRC Cambridge Stem Cell Institute, University of Cambridge, Cambridge, UK; 11 University of Edinburgh and the Autism Research Centre, University of Cambridge, Cambridge, UK; 12 Department of Clinical and Biomedical Sciences, University of Exeter Medical School, University of Exeter, Barrack Road, Exeter, UK; 13 Meir Medical Center; Tel Aviv University, School of Medicine, Tel Aviv, Israel; 14 Loke Centre for Trophoblast Research, Department of Physiology, Development and Neuroscience, University of Cambridge, Cambridge, UK; 15 University of Exeter Medical School, College of Medicine & Health, University of Exeter, Exeter, UK; 16 Methods of Plasticity Research, Department of Psychology, University of Zurich, Zurich, Switzerland; 17 Donders Institute for Brain, Cognition, and Behavior, Radboud University Nijmegen, Nijmegen, The Netherlands; 18 Department for Cognitive Neuroscience, Radboud University Medical Center, Nijmegen, The Netherlands; 19 Child Development Division, Maccabi Health Services, Tel Aviv, Israel; 20 Department of Neurology, University of California, Los Angeles, CA USA; 21 Department of Human Genetics, University of California, Los Angeles, CA USA; 22 Center for Autism Research and Treatment, Semel Institute, David Geffen School of Medicine, University of California, Los Angeles, CA USA; 23 Program in Neurobehavioral Genetics, Semel Institute, David Geffen School of Medicine, University of California Los Angeles, Los Angeles, CA USA; 24 Department of Psychology, University of Oslo, Oslo, Norway; 25 Nic Waals Institute, Lovisenberg Diaconal Hospital, Oslo, Norway; 26 PsychGen Center for Genetic Epidemiology and Mental Health, Norwegian Institute of Public Health, Oslo, Norway; 27 Maternal and Fetal Health Research Centre, School of Medical Sciences, University of Manchester, Manchester, UK; 28 Saint Mary’s Hospital, Manchester University NHS Foundation Trust, Manchester, UK; 29 Centre for Addiction and Mental Health, Campbell Family Mental Health Research Institute, Toronto, ON Canada; 30 Department of Psychiatry, The Hospital for Sick Children, Toronto, ON Canada; 31 Department of Psychiatry, Temerty Faculty of Medicine, University of Toronto, Toronto, ON Canada; 32 Department of Psychology, Faculty of Arts and Science, University of Toronto, Toronto, ON Canada; 33 Department of Psychiatry, National Taiwan University Hospital and College of Medicine, Taipei, Taiwan; 34 MRC Laboratory of Molecular Biology, Cambridge Biomedical Campus, Francis Crick Avenue, Cambridge, UK; 35 Laboratory for Autism and Neurodevelopmental Disorders, Center for Neuroscience and Cognitive Systems @UniTn, Istituto Italiano di Tecnologia, Rovereto, Italy; 36 Cell Division and Cancer group, Spanish National Cancer Research Centre (CNIO) Madrid, Madrid, Spain; 37 Human Embryo and Stem Cell Laboratory, The Francis Crick Institute, London, NW1 1AT UK; 38 Epigenetics Programme, Babraham Institute, Cambridge, CB22 3AT UK; 39 Department of Basic and Clinical Neuroscience, Institute of Psychiatry, Psychology and Neuroscience, King’s College London, London, UK; 40 MRC Centre for Neurodevelopmental Disorders, King’s College London, London, UK; 41 Social and Affective Neuroscience Group, IMT School for Advanced Studies Lucca, Lucca, Italy; 42 Department of Pediatrics, University of Cambridge, Cambridge, UK; 43 Faculty of Life Sciences and Medicine, King’s College London, London, UK; 44 Psychology Department, Ben-Gurion University, Beer-Sheba, Israel; 45 Danish Center for Neonatal Screening, Department of Congenital Disorders, Statens Serum Institut, Copenhagen, Denmark; 46 Aarhus University Hospital—Psychiatry, Psychosis Research Unit, Aarhus, Denmark; 47 NCRR, National Centre for Register-Based Research, Aarhus University, Aarhus, Denmark; 48 CORE - Copenhagen Research Center for Mental Health, Mental Health Centre Copenhagen, Copenhagen University Hospital, Copenhagen, Denmark; 49 Department of Clinical Medicine, Faculty of Health and Medical Sciences, University of Copenhagen, Copenhagen, Denmark; 50 Institute of Biological Psychiatry, Mental Health Services, Copenhagen University Hospital, Roskilde, Denmark

**Keywords:** Autism spectrum disorders, Genetics, Neuroscience

## Abstract

Genetic variants linked to autism are thought to change cognition and behaviour by altering the structure and function of the brain. Although a substantial body of literature has identified structural brain differences in autism, it is unknown whether autism-associated common genetic variants are linked to changes in cortical macro- and micro-structure. We investigated this using neuroimaging and genetic data from adults (UK Biobank, N = 31,748) and children (ABCD, N = 4928). Using polygenic scores and genetic correlations we observe a robust negative association between common variants for autism and a magnetic resonance imaging derived phenotype for neurite density (intracellular volume fraction) in the general population. This result is consistent across both children and adults, in both the cortex and in white matter tracts, and confirmed using polygenic scores and genetic correlations. There were no sex differences in this association. Mendelian randomisation analyses provide no evidence for a causal relationship between autism and intracellular volume fraction, although this should be revisited using better powered instruments. Overall, this study provides evidence for shared common variant genetics between autism and cortical neurite density.

## Introduction

Autism is a highly polygenic, heritable set of neurodevelopmental conditions, characterised by restrictive repetitive behaviours and unusually narrow interests, sensory hyper- and hypo-sensitivities, and social-communication difficulties. Twin and familial recurrence studies indicate that heritability of autism is as high as 60–90% [1, 2], although single nucleotide polymorphism (SNP) heritability estimates are more modest, ranging from 11–50% [3, 4].

It is unclear how the polygenic likelihood for autism gives rise to the cognitive and behavioural outcomes that are collectively referred to as autism. In line with our theoretical understanding that autism emerges from changes in brain structure and function, genetic studies have demonstrated an enrichment for autism-related genes and genetic variants in early neurodevelopmental processes [3, 5–8]. Furthermore, studies have identified differences in cortical morphology (structural and diffusion phenotypes) and functioning in autistic compared to non-autistic individuals (e.g., [9–11]) and among autistic individuals (e.g., [12–17]). Studies have also identified associations between brain morphology and autistic traits in the general population (e.g., [18, 19]).

Previous work has demonstrated that at least a subset of autistic individuals shows differences in cortical volumes and surface area (SA) during development. For instance, longitudinal scans of autistic and non-autistic children have identified cortical enlargement in autistic children after the first year of life [20, 21]. Additionally, common genetic variants associated with typical differences in surface area and volume are enriched in genes identified from rare variant studies of neurodevelopmental conditions, including autism [22].

A small number of studies have investigated the neural correlates of common genetic variants associated with autism, indexed by polygenic scores (PGS). These studies demonstrated that PGS for autism is associated with global and regional alterations in cortical volume, cortical thickness (CT), and surface area (SA) [18, 23], with one study demonstratign that the association between PGS for autism and CT varies with age [24]. However, these studies were conducted in fewer than 2500 individuals, which may be underpowered to detect small effect sizes using PGS for autism. A larger study in the UK Biobank demonstrated that PGS for autism is associated with multivariate differences in brain asymmetry [25] and with differences in brain white-matter connectivity [26].

Currently, there are no large-scale studies that systematically investigate how PGS for autism is associated with structural and diffusion derived brain imaging measures. We hypothesised that common genetic variants associated with autism may also be associated with a range of neuroimaging phenotypes, in both adults and children. In particular, we were interested in the role of autism-associated common genetic variants in measures generated from Diffusion Tensor Imaging (henceforth, cortical microstructure in line with refs. [27–29]) given previous associations between cortical microstructure and genetic variation for schizophrenia and depression [30–32].

In parallel, studies have identified substantial sex differences in both brain structure [33–36] and the presentation and diagnosis of autism [37, 38]. Notably, even after accounting for social processes, there still is a male preponderance of autism diagnosis [38], suggesting that there may be biological factors that contribute to sex differences in autism. For instance, one study has demonstrated a shift in multivariate neuroanatomical patterns in autistic individuals towards that typically observed in males [33]. Subsequently, we were also interested in investigating if sex differences in autism are reflected in the sex differential effects of PGS for autism on brain structure.

**Fig. 1:**
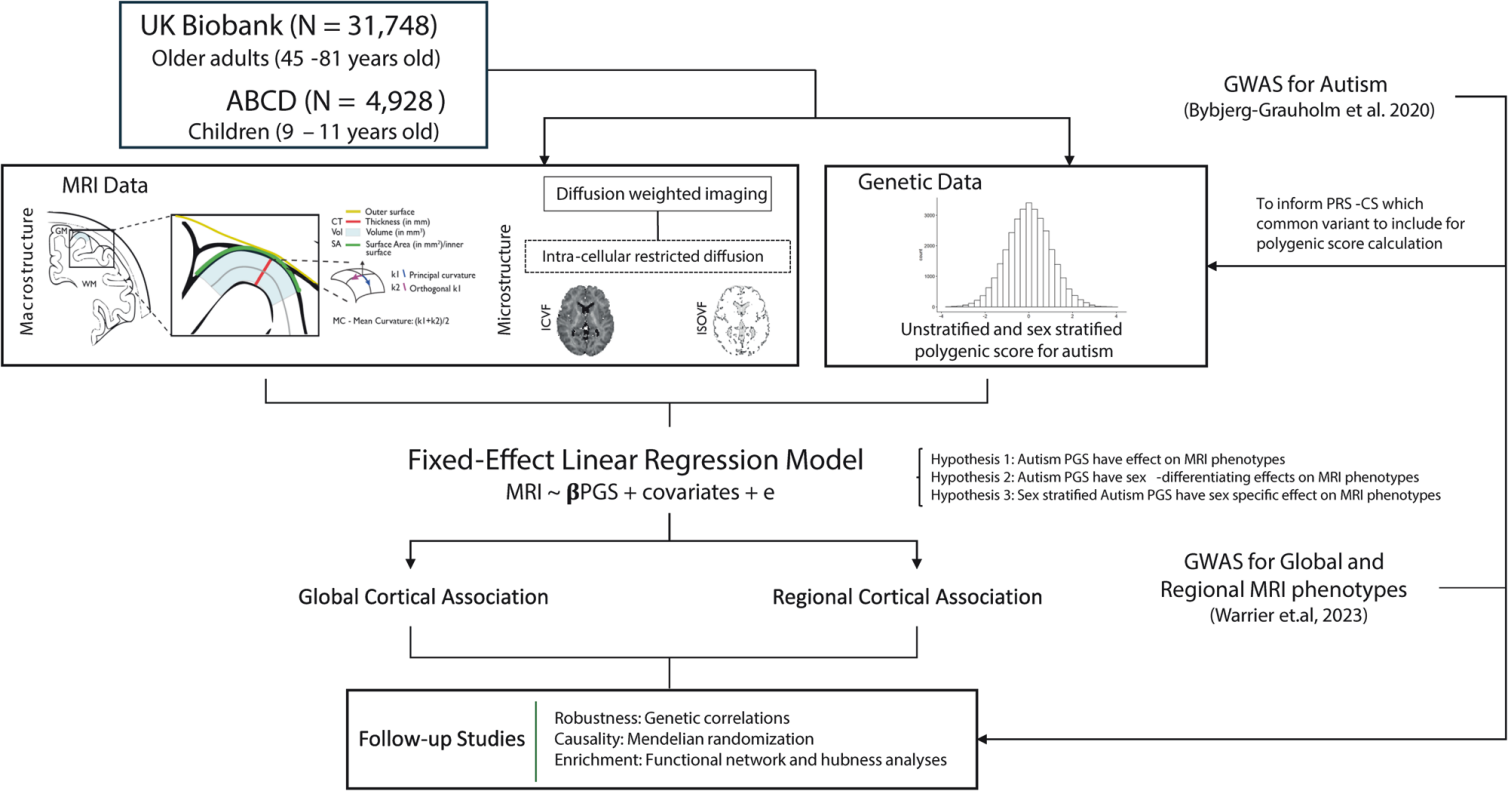
Schematic summary. We generated polygenic scores for autism in two datasets - UK Biobank and ABCD and investigated the associations between the polygenic scores and five brain structural phenotypes globally as well as in 180 regions. We fitted sex-stratified and unstratified models. We confirmed the robustness of the results using genetic correlations and assessed causality using Mendelian randomization. Finally, to contextualise the results, we investigated enrichment of the associations in cortical networks.

Here, we address these questions using the largest homogenous neuroimaging genetics datasets available in adults (UK Biobank, N = 31,748) and children (ABCD, N = 4928). We investigated the association between genetic likelihood for autism and variation in brain structure at a global and regional level in the general population, as well as potential sex differences in this association (Fig. 1). We studied three macrostructural and two microstructural MRI-derived phenotypes both globally as well as 180, bilaterally averaged cortical regions based on the Glasser parcellation. We focus on these five phenotypes, as these five are highly correlated with other cortical phenotypes, index five different latent traits, and have relatively high SNP heritability compared to several other measurable phenotypes [22]. Follow-up analyses such as genetic correlation and Mendelian randomisation were conducted to investigate the robustness of the results and causality, respectively. Finally, to contextualise the results, we also ran enrichment analyses for the autism PGS association with known functional networks [39] and cytoarchitectonic classes [40].

## Methods

### Participants

This study is based on data from two independent cohorts, the UK Biobank (UKB) [41] and the Adolescent Brain Cognitive Development (ABCD) database [42]. In both cohorts, we excluded participants with incomplete MRI and genotype data and focused on participants with genetically inferred European ancestry. This generated two subgroups, consisting of N = 31,748 UKB participants and N = 4928 ABCD participants. Details of age, sample size, and sex are summarised in Supplementary Table (ST) 1. Ethical approval for analysing de-identified data was obtained from the Human Biology Research Ethics Committee, University of Cambridge (Cambridge, UK). Informed consent was provided by all participants.

### Acquisition, preprocessing and quality control for imaging data

For MRI data, minimally preprocessed T1 and T2-FLAIR weighted data were downloaded from UKB (application 20904) and ABCD through their respective data repository, and further processed using FreeSurfer version (Version 6.0.1) ([43]) for cortical reconstruction [22].

Then, the data was parcellated using the Human Connectome Project parcellation (i.e. Glasser parcellation) [44] using surface-to-surface mapping to align the diffusion and T1-weighted imaging. The recon-all reconstruction used both T1- and T2- weighted images when both were available, and all subsequent statistical analyses included a covariate for the type of reconstruction (i.e., T1 only or T1-T2 combined). Reconstruction quality was evaluated using the Euler index [45] and was included as a covariate in subsequent statistical analyses. Macrostructural MRI-derived phenotypes, including total surface area of the cortex (SA), average cortical thickness (CT), and total mean curvature (MC) were extracted and standardised for further analysis.

Reconstruction of Neurite orientation dispersion and density imaging (NODDI) was generated by the Accelerated Microstructure Imaging via Convex Optimization (AMICO) pipeline [27] and was then aligned to the Glasser parcellation. NODDI can measure three types of microstructural environment in the human brain. In this study, we used two of them: a measure of the density neurites including axon and dendrites by measuring the extent of intracellular diffusion (Intracellular Volume Fraction or ICVF) and a measure of isotropic volume, typically thought to represent free-water or the cerebrospinal fluid (Isotropic Volume Fraction or ISOVF). ICVF is also called Neurite Density Index (NDI).

Global values of the MRI-derived phenotypes were obtained by averaging or summing all values from the 360 regions. Regional values of MRI-derived phenotypes were bilaterally averaged, resulting in 180 regional values. Outliers were removed by excluding individuals with more than 5 standard deviations (SD) or median absolute deviations beyond the mean and median respectively.

In addition, as previously conducted [31], probabilistic tractography was used to calculate ICVF values at each of 15 major white matter tracts defined using AutoPtx [46].

Finally, to compare the findings from the NODDI microstructural metrics, as sensitivity analyses we ran additional analyses using more widely used metrics of microstructure - Mean Diffusivity (MD) and Fractional Anisotropy (FA) in both the UKB and ABCD.

### Acquisition and quality control for genetic data

Details of the genome-wide genotype data used for this study including processing, imputation, and quality control can be found in detail elsewhere for UKB [47] and ABCD [22, 48].

Briefly, in both datasets, we excluded participants who were not of genetically inferred European ancestry based on self-reported data and genetic principal component-based clustering. From the group of included participants, we further excluded individuals with less than 95% genotyping rate, who had a mismatch between genetic and reported sex, and who had excess genetic heterozygosity. Additionally, we also excluded any individuals who were more than ±5 standard deviations from the means of the first two genetic principal components to minimise the impact of fine-scale population stratification on the analyses.

In both UKB and ABCD, we included genotyped and imputed SNPs with a minor allele frequency >0.1%, Hardy-Weinberg equilibrium (P > 1 × 10^−6^), genotyping rate >95%, and, for imputed SNPs, imputation quality R^2^ > 0.4.

### Polygenic scores

Unstratified and sex-stratified polygenic scores (PGS) for autism were calculated for UKB and ABCD using PRS-CS [49], based on effect sizes of autosomal SNPs provided by autism GWAS summary statistics from the iPSYCH cohort [50]. We used the default gamma-gamma priors, and set the global shrinkage prior (“phi”) to 1e-2. We used the 1000 Genomes European subsample LD reference panel provided by PRScs.

The iPSYCH GWAS consists of 19,870 autistic participants (15,025 males) and 39,078 non-autistic individuals (19,763 males). All PGS were standardised to have a mean of zero and a standard deviation of one. We used the iPSYCH GWAS compared to the publicly available Grove et al., 2019 GWAS [3] because the iPSYCH had a larger sample size, better statistical power (mean X^2^ = 1.23 for iPSYCH GWAS and 1.2 for Grove et al., 2019), is unlikely to be affected by participation bias, and because we had access to sex-stratified GWAS data from the same sample.

PGS for the iPSYCH autism GWAS explained 0.28% (p = 4.19 × 10^−3^) of the variance in autism diagnosis in a sample of 370 autistic individuals and 9992 randomly selected non-autistic individuals in the UKB. As expected, autistic individuals had higher autism PGS compared to non-autistic individuals. There were fewer than 100 autistic individuals in ABCD and we did not have sufficient statistical power to investigate the variance explained by the autism PGS.

### Statistical analyses

All statistical analyses between PGS for autism and MRI-derived phenotypes were conducted in R (Version 4.3.1).

We first investigated whether PGS for autism is associated with variation in MRI-derived global and regional phenotypes (Hypothesis 1, Eqs. 1 and 2). To identify the effect of PGS for autism on region-specific effects, global values of MRI-derived phenotypes of interest were included as covariates in conditional analyses (Eqs. 3, 5, 7 and 9).

Given that the mean scores of the MRI-derived phenotypes differed between sexes (ST 2), for sex differential effects, we investigated if PGS for autism is differentially associated with variation in MRI-derived phenotypes by sex by including a sex x PGS interaction term into the model (Hypothesis 2). We tested this across both global and regional phenotypes (Eqs. 4–5). The genetic correlation between the two sex-stratified autism GWAS, although high, was significantly less than 1. Given this, we also investigated whether sex-stratified PGS for autism will have sex-specific effects on MRI-derived phenotypes in males and females separately both globally and regionally (Hypothesis 3, Eqs. 6–9).

For all regressions we included age, age^2^, the first 10 genetic principal components, genotype sequencing batch, scanning site number, Euler index, framewise displacement [22], and T1-T2 scan status. T1-T2 status refers to whether individuals were preprocessed with FreeSurfers recon-all pipeline using either the T1 weighted image only, or were processed using the combined T1 and T2 to improve reconstruction. The choice of processing pipeline was purely determined by the availability of the relative T2 weighted image.

These covariates were added to minimise the effect of confounding variables, based on availability within the cohort database. For Hypothesis 1 we further included sex, sex x age, and sex x age^2^ as covariates.

All genetic and imaging data were standardised before and after sex stratification. All continuous technical covariates, such as Euler Index were also similarly standardised. For all associations with regional MRI-derived phenotypes, the p values were adjusted for multiple testing for the total number of Glasser parcellation regions for each hemisphere (n = 180) using Benjamini-Hochberg false discovery rate (FDR) [51]. We used an FDR-corrected p value ≤ 0.05 as the statistical significance threshold.

#### Equations underlying the Linear Regression models

**Hypothesis 1:** Autism PGS is associated with variations in MRI-derived phenotypes:

1 $${Glb}.{MRI} \sim 	\,{PGS}+{Sex}+{Age}+{{Age}}^{2}+{Sex}\!\!:{Age}+{Sex}\!\!:{{Age}}^{2}\\ 	+{Technical\; Covariates}$$

2 $${Reg}.{MRI} \sim 	\,{PGS}+{Sex}+{Age}+{{Age}}^{2}+{Sex}\!\!:{Age}+{Sex}\!\!:{{Age}}^{2}\\ 	+{Technical\; Covariates}$$

3 $${Reg}.{MRI} \sim 	\,{PGS}+{Glb}.{MRI}+{Sex}+{Age}+{{Age}}^{2}+{Sex}\!\!:{Age}\\ 	+{Sex}\!\!:{{Age}}^{2}+{Technical\; Covariates}$$

**Hypothesis 2**: Autism PGS has sex-differential association with MRI-derived phenotypes:

4 $${Glb}.{MRI} \sim 	\,{PGS}+{PGS}\!\!:{Sex}+{Sex}+{Age}+{{Age}}^{2}+{Sex}\!\!:{Age}\\ 	+{Sex}\!\!:{{Age}}^{2}+{Technical\; Covariates}$$

5 $${Reg}.{MRI} \sim 	\,{PGS}+{PGS}:{Sex}+{Glb}.{MRI}* +{Sex}+{Age}\\ 	+{{Age}}^{2}+{Sex}:{Age}+{Sex}:{{Age}}^{2}+{Technical\; Covariates}$$

**Hypothesis 3:** Sex stratified autism PGS have sex-specific associations with MRI-derived phenotypes:

6 $$f.{Glb}.{MRI} \sim {fPGS}+{Age}+{{Age}}^{2}+{Technical\; Covariates}$$

7 $$f.{Reg}.{MRI} \sim {fPGS}+f.{Glb}.{MRI}* +{Age}+{{Age}}^{2}+{Technical\; Covariates}$$

8 $$m.{Glb}.{MRI} \sim {mPGS}+{Age}+{{Age}}^{2}+{Technical\; Covariates}$$

9 $$m.{Reg}.{MRI} \sim {mPGS}+m.{Glb}.{MRI}* +{Age}+{{Age}}^{2}+{Technical\; Covariates}$$

Note: MRI indicates the MRI-derived phenotype of interest. Prefix Glb. indicates global MRI-derived phenotype value; prefix Reg. indicates Regional MRI-derived phenotype; prefix f. indicates female-stratified values; prefix m. indicates male-stratified values. For Eqs. 5, 7 and 9, we ran separately with and without the global MRI-derived phenotypes as covariates. This is indicated by Glb.MRI*.

### Bidirectional Mendelian randomization analyses and genetic correlations

We investigated the causal relationship between autism and the global and regional imaging phenotypes using two-sample bidirectional Mendelian randomization (MR) analyses, using the ‘TwoSampleMR’ (Version 0.5.7) and ‘MRPRESSO’ (Version 1.0) packages in R. Specifically, we were interested in understanding if (i) autism (exposure) causes changes in brain structure indexed by MRI-derived phenotypes (outcome) and/or (ii) changes in brain structure indexed by MRI-derived phenotypes (exposure) causes autism (outcome). For autism, we used SNPs from the unstratified autism GWAS from iPSYCH. For MRI-derived phenotypes, we used the meta-analysed GWAS of ABCD and UKB [22]. For all GWAS of exposure phenotype, we included only SNP genetic instruments that reached genome-wide significance (p < 5 × 10^−8^), after clumping at 10,000 kb distance and an LD r^2^ of 0.001.

We fitted different MR models, including inverse variance weighted (IVW) MR, median weighted (majority valid), MR Egger (accounts for pleiotropy,) and MR PRESSO (detects and excludes outliers in the instrument) to test the robustness of the results. We conducted further sensitivity analyses, such as testing for heterogeneity and horizontal pleiotropy, and leave-one-out to interrogate the validity of the results.

Genetic correlations between autism and global and regional MRI-derived phenotypes were estimated through LDSC (Version 1.0.1) [52], using Autism GWAS summary statistics from the iPSYCH cohort [50] and meta-analysed GWAS summary statistics of each MRI-derived phenotypes from the combined ABCD and UKB cohorts [22]. GWAS was run separately for both cohorts and then meta-analysed. The summary statistics for the MRI-derived phenotypes were obtained from a previous study that conducted GWAS in 36,663 individuals from the UKB and ABCD by the current team. Further details are available in ref. [22], but briefly, GWAS was conducted on standardised MRI phenotypes using a similar genetic and phenotypic quality control procedure as outlined here, using fastGWA. For all GWAS, age, age^2^, sex, age × sex, age^2^ × sex, imaging center, first 40 genetic principal components, mean framewise displacement (as obtained from the accompanying resting-state fMRI scan), maximum framewise displacement (as obtained from the accompanying resting-state fMRI scan) and Euler Index were included as covariates. In addition, for structural MRI metrics derived from T1, we included the inclusion of T2 scans as covariates as this influenced the calculation of these metrics.

The LD score used is provided by the North-West European population from the 1000 Genomes project [53]. For genetic correlation between autism and regional MRI-derived phenotypes, only regions that were significant in Eq. 2 were included in the analysis. All results were corrected for multiple corrections using Benjamini Hochberg False Discovery Rate (FDR) corrected p value ≤ 0.05.

### Network and enrichment analyses

We ran a series of analyses to understand the links between brain networks and PGS for autism.

Conceptualising the brain as a connected network, we generated hubness scores for each region by calculating both mean phenotypic (structural connectivity hubness) and genetic (genetic hubness) correlation [54] between each cortical region and every other cortical region.

Specifically, structural connectivity hubs were calculated using phenotypic data separately in the UKB and ABCD, where a 180 × 180 pairwise correlation matrix was constructed for all 180 regional phenotypes. The hubness score for each region is then generated by averaging the Pearson’s coefficient of each region.

For genetic hubness we used the genetic correlation values generated using LDSC from the meta-analysed GWAS of ABCD and UK Biobank [22]. Similar to structural connectivity, the genetic hubness of each region was then estimated by averaging the genetic correlation for each region. The derived genetic hubness score also ranges between [0,1]. As the hubness score is close to 1, it means on average more regions are strongly and/or positively correlated to the alterations happening with the region of interest. We then investigated the correlation between hubness scores and PGS association across regions.

We further investigated if the association of PGS with the regional phenotypes were enriched in cortical networks identified from intrinsic functional connectivity (Yeo-Kreinen networks [39]) and cytoarchitectonic classes (Mesulam classes [40]). Enrichment analyses were conducted using 1000 “spin” permutation testing [55], which accounts for the spatial autocorrelation among regions. Results were FDR-corrected for each MRI-derived phenotype and cortical atlas.

### Sex-stratified GWAS of global imaging phenotypes

To investigate potential sex differences, we conducted GWAS of global SA, CT, MC, ICVF and ISOVF in the UK Biobank separately in males (N = 14,957) and females (N = 16,833). We followed the same pipeline as used before [22] and genetic quality control is detailed earlier in the section “Acquisition and quality control of genetic data”. All phenotypes were standardised, and we included the same covariates included in the PGS analyses to test Hypothesis 1. GWAS was conducted using fastGWA [56] which uses a linear mixed effects model to account for fine-scale population stratification and relatedness among participants. We used the sex-stratified GWAS for genetic correlation analyses with the sex-stratified autism GWAS to investigate sex-specific effects.

## Results

### Autism polygenic scores are associated with reduced global cortical ICVF

**Fig. 2:**
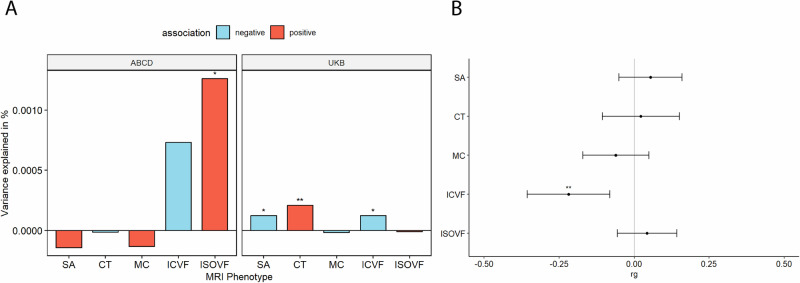
Statistical and Genetic correlation between polygenic scores for autism and global MRI-derived phenotypes. **A** Percentage of variance explained by polygenic score for autism (PGS) for different global cortical MRI-derived phenotypes under cohorts. SA surface area, CT cortical thickness, MC mean curvature, ICVF intracellular volume fraction or neurite density index, ISOVF isotropic volume fraction. Blue bar indicates negative associations, and the red bar indicates positive associations. * p <= 0.05, ** p <= 0.01. **B** Genetic correlation (rg) between autism and MRI-derived phenotype GWAS. Whiskers indicate 95% confidence intervals. Asterisks indicate p values after FDR<5% correction, with code sign the same as A.

We first investigated the association between PGS for autism and five global (standardised summed or averaged) structural or diffusion MRI-derived phenotypes in both the UK Biobank and ABCD. These include surface area (SA), cortical thickness (CT), mean curvature (MC), Intracellular Volume Fraction (ICVF, also called Neurite Density Index or NDI), and Isotropic Volume Fraction (ISOVF). We chose these five phenotypes as they are highly correlated with other structural and diffusion phenotypes representing five cortical latent traits, have low correlations between each other, and have higher SNP heritability compared to other highly correlated phenotypes [22]. In the UKB, PGS for autism was significantly associated with lower SA (Incremental variance explained by the PGS i.e., R^2^: 1.23e-04) and ICVF (R^2^: 1.23e-04), and increased CT (R^2^: 2.10e-04) after FDR correction (Fig. 2A and ST 3). In the younger ABCD cohort, higher PGS for autism was nominally significantly associated with lower ICVF (R^2^: 7.31e-04) and significantly with higher ISOVF (R^2^: 1.26e-03) (ST 3). We further confirmed the robustness of these observations through genetic correlation, where we observed a significant negative genetic correlation between ICVF and autism (r_g_ = −0.22, s.e.m = 0.07, p = 1.80e-3) (Fig. 2B and ST 4).

### PGS for autism are negatively associated with regional cortical ICVF

**Fig. 3:**
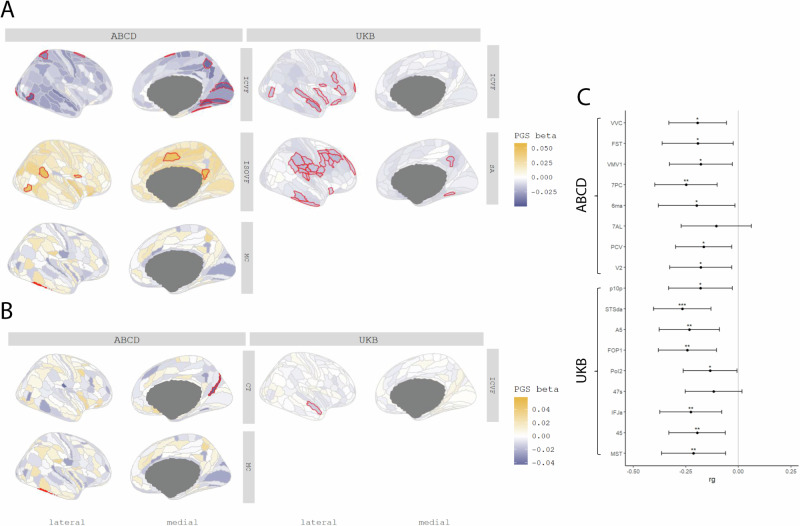
Statistical and Genetic correlation between polygenic scores for autism and regional MRI-derived phenotypes. **A** Cortical map of regional associations between PGS for autism and cortical MRI-derived phenotypes. Red outline means the effect of PGS for autism reached statistical significance at FDR <=0.05. **B** Cortical map of regional associations between PGS for autism and cortical MRI-derived phenotypes after global phenotype correction. Red outline means the effect of PGS for autism reached statistical significance at FDR <=0.05. For brevity, we have displayed only regional association maps for phenotypes where there was at least one significant regional association. **C** Genetic correlation between autism and regional ICVF in regions with statistically significant associations with the PGS for autism. Asterisks indicate p values after FDR correction: * p <= 0.05, ** p <= 0.01, *** p <= 0.001.

We next investigated whether PGS for autism are associated with regional variation in the same five MRI-derived phenotypes. In the UKB, we identified a negative association between PGS for autism and ICVF in nine regions (Inc.R^2^: 2.25e-4 to 5.72e-4) and with SA in 24 regions (Inc.R^2^: 1.53e-4 to 3.60e-4) after FDR correction (Fig. 3A). We did not identify a significant association with any other regional phenotypes (ST 5).

In ABCD, despite the smaller sample size, ICVF was significantly negatively associated with PGS for autism in eight regions (Inc.R^2^: 1.64e-3 to 2.11e-3) (Fig. 3B), although these regions differed from the significantly associated regions in the UKB (ST 6). In the UKB, the significant associations with ICVF were primarily in the frontal and temporal lobes whereas in ABCD, the significant associations were in the occipital lobe. In addition, PGS for autism was positively associated with MC (Inc.R^2^: 3.20e-3) in the lateral temporal region, and ISOVF in five regions (Inc.R^2^: 1.96e-3 to 2.36e-3).

Supporting the robustness of the associations with ICVF, autism had significant and negative genetic correlations with ICVF in 15 of the 17 regions that were significantly associated with autism PGS (ST 4 and Fig. 3C). In contrast, we did not identify any significant associations between autism and SA, MC and ISOVF. Together, these analyses identify robust shared genetics between autism and ICVF using two different methods and in two different cohorts.

To account for the effect of the global phenotypes, we re-ran the analyses after correcting for the global phenotypes. In these conditional analyses, ICVF in one region, STSda (Inc.R^2^: 2.26e-4) located in the superior temporal gyrus, and MC in TE2P, located in the fusiform gyrus (Inc.R^2^: 3.19e-3), remained significantly associated with PGS for autism. We additionally found one region in CT named DVT, located in the posterior cingulate, which was significantly associated with PGS for autism (ST 7 and ST 8).

### Autism PGS is more negatively associated with ICVF in cortical hubs

**Fig. 4:**
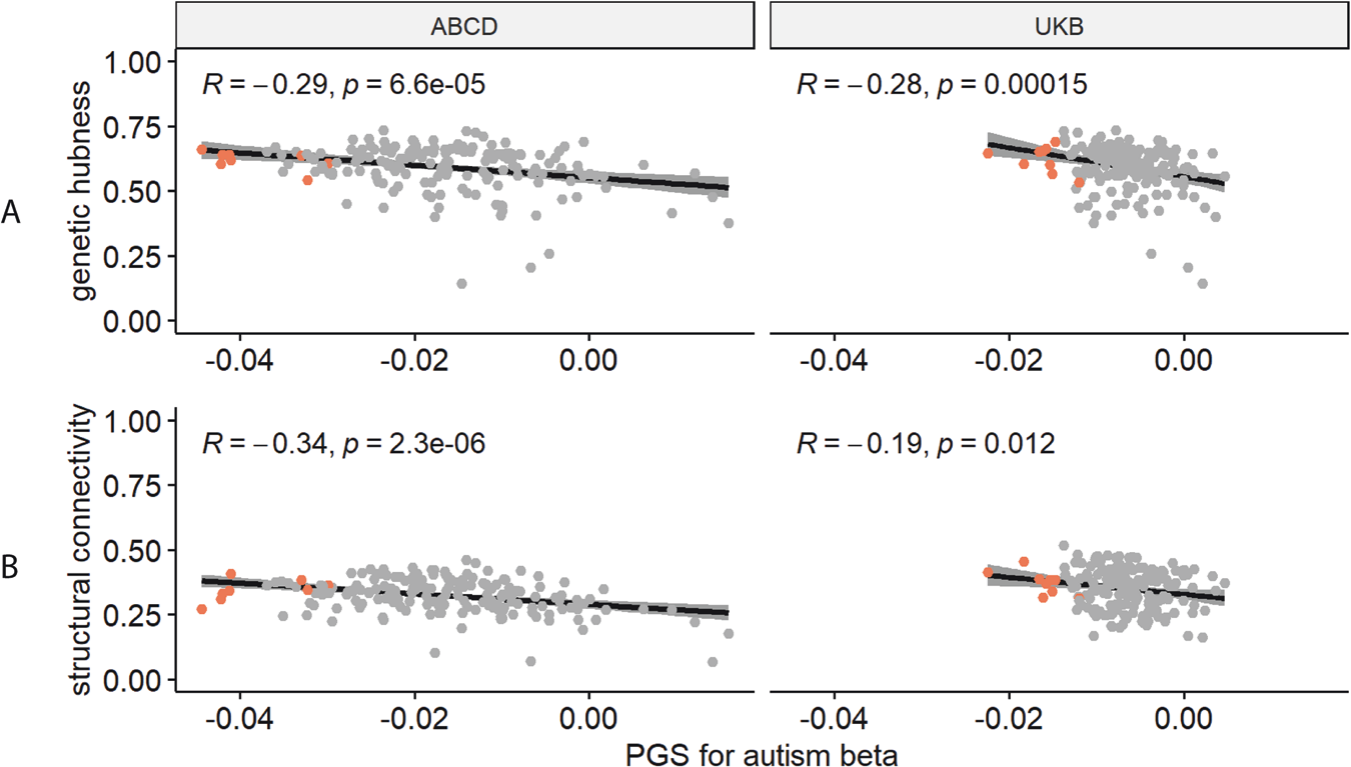
Enrichment Analysis. **A** Correlation plot between genetic hubness and autism PGS association with ICVF in the ABCD and UK Biobank cohorts. **B** Correlation plot between structural connectivity and autism PGS association with ICVF in the ABCD and UK Biobank cohorts. Each point represents a region and regions where there is a significant association between the phenotype and autism PGS are coloured in red. R is the Pearson correlation coefficient. p is the p value before correction for multiple testing, all p-value remained significant (<0.05) after FDR correction.

We recognise that cortical regions are not truly independent but may be organised as networks based on structural or genetic similarity, or functional co-activation [39], or cellular similarity [40]. For example, CT in regions that co-vary or are connected with several other regions (termed hubs) were more likely to be altered in autism [57]. Investigating this hypothesis, we observe a significant correlation between genetic hubness and PGS association with autism for ICVF in both the UKB and ABCD, and CT in ABCD (ST 9, Fig. 4). Further supporting this, and in line with Cheverud’s conjecture, we identified a significant correlation between structural connectivity and PGS association for ICVF in both ABCD and UKB. We also identified a significant association between structural connectivity and autism PGS association with CT in the ABCD and ISOVF in the UKB.

In addition to the significant association between hubness and ICVF, regions with statistically significant associations with autism PGS also on average had higher hubness (Fig. 4, Supplementary Figures 1 and 2). We further considered if there is an association of the PGS effects in known functional networks (Yeo) or cytoarchitectural classes of the cortex (Mesulam). After spin permutation correction, PGS associations for MC were enriched in the idiotypic cortex (Mesulam class) in both ABCD and UKB (ST 10). We did not identify any other robust enrichments across both ABCD and UKB.

### PGS for autism are negatively associated with ICVF in white matter tracts

**Fig. 5:**
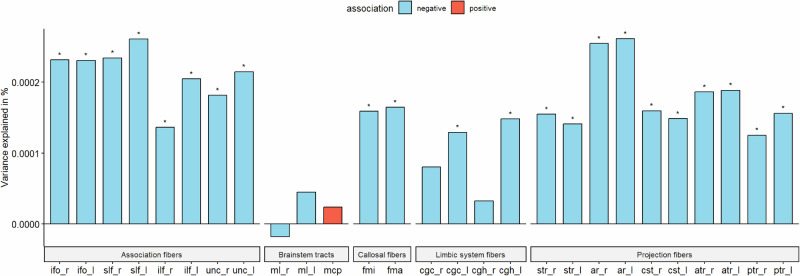
White matter tract association. Percentage of variance explained by polygenic score for autism (PGS) for 15 major white matter tracts for ICVF in UKB. Blue bar indicates negative associations, and the red bar indicates positive associations. * p <= 0.05. The acronyms for the 15 major white matter tracts are mcp = middle cerebellar peduncle, ml = medial lemniscus, cst = corticospinal tract, ar = acoustic radiation, atr = anterior thalamic radiation, str = superior thalamic radiation, pts = posterior thalamic radiation, slf = superior longitudinal fasciculus, ilf = inferior longitudinal fasciculus, ifo = inferior fronto-occipital fasciculus, unc = uncinate fasciculus, cgc = cingulate gyrus part of cingulum, cgh = parahippocampal part of cingulum, fmi = forceps minor, and fma = forceps major. Suffix _r means it is estimated from the right hemisphere and _l means it is estimated from the left hemisphere.

As PGS for autism consistently showed significant negative associations with cortical ICVF, we were interested in investigating if similar effects would be observed for 27 white matter tracts. We investigated this only in the UKB due to data availability. PGS for autism was significantly negatively associated with ICVF in 22 out of 27 ICVF white matter tracts (R^2^ from −1.77e-05 to 2.15e-04) (ST 11 and Fig. 5). The variance in white matter tracts ICVF explained by the autism PGS was similar to that of regional cortical ICVF in the UKB. However, in contrast to associations with cortical ICVF, we did not observe significant genetic correlations between autism and ICVF in white matter tracts (ST 4).

### No evidence for causal relationships between autism and MRI-derived phenotypes using bi-directional Mendelian randomisation

Since there were significant genetic correlations between PGS for autism and global and regional MRI-derived phenotypes, we additionally investigated whether there is a casual relationship between autism and these MRI-derived phenotypes or vice versa using four different Mendelian randomisation methods. Specifically, we focussed on the phenotypes (global and regional) where we observed a significant association with PGS, hypothesising that causal relationships will lead to shared genetics. Additionally, for regional phenotypes, we excluded any phenotype with fewer than three genome-wide significant SNPs after harmonisation.

For global MRI-derived phenotypes, after adjusting for multiple correction based on MRI-derived phenotype tested, we found no significant evidence for causal effect of MRI-derived phenotypes on autism, nor causal effect of autism on MRI-derived phenotypes across four MR methods tested (ST 12 and 13). Similar results were also seen for autism and regional MRI-derived phenotypes after multiple testing corrections (ST 14 and 15).

### Limited evidence for sex differential effects of autism PGS on brain structure

Given that more males are diagnosed as autistic compared to females, we investigated if some of the sex differences in both the manifestation and diagnosis of autism are attributable to sex-differential effects in the cortex.

**Fig. 6:**
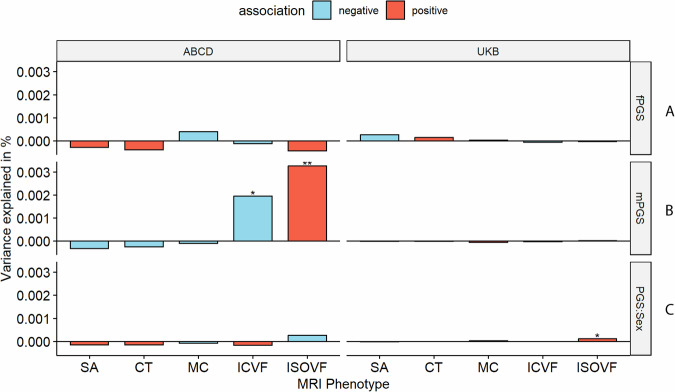
Percentage of variance explained by sex-specific and sex-differentiating PGS for autism. **A** and **B** Percentage of variance explained by sex stratified autism PGS for the global phenotypes in the equivalent sexes in ABCD and UKB. **C** Percentage variance explained by the sex interaction analysis. Asterisks indicate P values after FDR correction: * P <= 0.05, ** P <= 0.01. mPGS = polygenic scores from the males-only GWAS, fPGS = polygenic scores from the females-only GWAS, and PGS:Sex = the interaction between sex and PGS. SA = surface area, CT = cortical thickness, MC = mean curvature, ICVF = intracellular volume fraction.

We first investigated if the effects of autism PGS on MRI-derived phenotypes are statistically different between sexes by including a PGS x sex interaction term (Fig. 6). For the global phenotypes, we found a significant sex by PGS interaction for ISOVF in UKB (ST 3), but this was not significant in ABCD (ST 3) nor generalisable to regional ISOVF phenotypes (ST 16 and ST 17). In ABCD, we found regional sex differential associations for CT in three regions only after global phenotype correction, although this was not significant in the UKB (ST 18 and ST 19). Overall, we did not find robust evidence to suggest sex differential effects of autism PGS on cortical MRI-derived phenotypes.

The GWAS for autism in males and females showed high correlation (r_g_ = 0.80, s.e.m = 0.08, p = 2.22e-20), but the correlation was still significantly less than 1. This suggests the possibility of sex-specific genetic influences on autism. To further investigate potential sex differences, we used the sex-stratified autism GWAS to generate sex-specific polygenic risk scores for autism. We then examined the effects of these sex-specific scores in individuals of the same sex in the UK Biobank and ABCD globally (ST 3 and Fig. 6) and regionally (ST 20 to ST 27). In UKB, we did not observe any sex-specific effect of PGS for autism. In ABCD, we observed significant male-specific autism PGS effects on ICVF and ISOVF in males (ST 3). Regionally, we found no male-specific, nor female-specific associations in UKB. In ABCD, we found male-specific regional effects for ICVF in one region (PCV in the precuneus) and ISOVF in nine regions. Additionally, after global phenotype correction, we identified male-specific regional effects for ISOVF in three regions. We did not find any significant genetic correlation between the sex-stratified autism GWAS and sex-stratified global MRI-derived phenotypes after multiple testing correction (ST 4).

### No association between autism PGS and either MD or FA

For the primary investigation we focussed on the association between autism PGS and cortical microstructure measured using the NODDI metrics of ICVF and ISOVF. However, these phenotypes are less widely used in the field. Subsequently, in post hoc analyses we conducted associations between autism PGS and mean diffusivity (MD) and fractional anisotropy (FA). We had previously established that MD is phenotypically and genetically correlated with ISOVF, and FA is phenotypically and genetically correlated with ICVF. However, in comparison to both ICVF and ISOVF, FA and MD have low SNP heritabilities [22]. Reflecting this low SNP heritability, we find no significant, replicable association between autism PGS and either FA or MD in both ABCD and UKB (ST 28–32). Nevertheless, the regression coefficients (i.e. the effect of the autism PGS on the regional phenotypes) were correlated between ICVF and FA (UKB : r = 0.52, 95% CI = 0.40–0.62, p = 1.85 × 10^−7^, ABCD: r = 0.37, 95% CI = 0.27–0.49, p = 4.09 × 10^−14^) and between ISOVF and MD (UKB: r = 0.67, 95% CI = 0.58–0.74, p < 2.2 × 10^−16^, ABCD: r = 0.17, 95% CI = 0.02–0.31, p = 0.019).

## Discussion

There has been a longstanding interest in how the genetic likelihood for autism manifests in the brain. Here, we investigate this by interrogating the correlates of common genetic variants for autism and MRI-derived brain phenotypes. In the largest sample to date, we systematically investigate the relationship between PGS for autism and MRI-derived phenotypes, beyond CT and SA [18, 24], and also investigate how sex and sex differences modify these relationships.

We found widespread negative associations between PGS for autism and ICVF, a measure of neurite density. ICVF, measured using the NODDI orientation sequence, is thought to be a measure of the density of neurites (axons and dendrites) in the brain, which has been supported by histological studies in humans [58] and mice [59]. This association was observed in both adults and children and validated using two different methods: polygenic scores and genetic correlations. This age-invariant association is noteworthy as neurite density changes non-linearly with age: it increases rapidly during childhood and decreases in later adulthood [60, 61]. One explanation of this robust association across ages is that the genetic likelihood for autism, alters the development of neurite density across development. Although Mendelian Randomisation did not identify a causal relationship, this could be due to the reduced statistical power of the instruments used. Alternatively, the shared genetics between autism and neurite density could emerge from pleiotropy or due to cross-trait assortative mating [62]. This association between autism and reduced neurite density is supported by previous case-control imaging studies [63–65] and mice studies focusing on autism-associated genes [66, 67]. These findings are paralleled by early postmortem brain studies in autism, which have identified cortical disorganisation [68] and reduced mini-columns in autism [69]. However, these findings have typically been conducted in small cohorts, and have not been consistently replicated. Furthermore, it is difficult to map microscale changes in neural cytoarchitecture, identified from postmortem studies, to relatively gross changes captured by MRI.

The association with reduced ICVF, at least globally, is not specific to autism, but also associated with many other traits and conditions, such as sleep, seizure, psychosis, schizophrenia, and Parkinson’s disease [31, 70, 71]. For instance, previous work has identified that PGS for schizophrenia is associated with reduction in global and regional ICVF [31]. However, the effects of the autism PGS on ICVF were only modestly correlated with the effects of schizophrenia PGS on ICVF (r = 0.33, 95% CI = 0.19–0.45), suggesting that although both autism and schizophrenia PGS are associated with reduction in ICVF, their effects vary. Notably, this is in line with the genetic correlation between autism and schizophrenia. It is unclear if reduction in ICVF is a transdiagnostic marker of neurodevelopmental and mental health conditions, or a marker of a co-occurring condition that is shared between autism and schizophrenia, for example, depression and anxiety.

The association with ICVF was widespread across the cortex and differed regionally between ABCD and the UKB. This may reflect age related changes in ICVF, age related genetic effects, or simply heterogeneity between cohorts. However, in both cohorts, for ICVF there was a strong correlation between hubness and the magnitude of association with autism PGS. One plausible explanation for this is the shared genetics between intracortical connectivity and autism. In other words, the same underlying mechanism that results in greater connectivity among regions may also contribute to variation in autistic traits. Similar findings have also been observed with schizophrenia [32, 72], suggesting broader shared genetics between determinants of hubness and neurodevelopmental and psychiatric outcomes.

In addition to ICVF, this study has also found significant global associations between PGS for autism, CT and SA in the adult study cohort, but not in the ABCD, possibly because of relatively small sample sizes leading to low statistical power to detect small effects. Large-scale case-control neuroimaging studies and meta-analysis have identified increased global CT and grey matter volume in autism [9, 11]. For regional phenotypes, beyond ICVF, this study observed significant associations between PGS for autism and SA for adults, associations between PGS for autism and MC for children. The lack of consistent findings across cohorts suggests that other factors, such as age difference between cohorts might also modify the association between autism and brain structure. The validity of these findings will require further testing using additional datasets and methods. Additionally, we also did not replicate previous regional findings in CT [24], potentially due to methodological differences or due to regression towards the mean [73].

For sex differences, we observed some sex-specific global associations between sex-stratified PGS and MRI-derived phenotype in sex-stratified sample populations, but no association was observed for regional phenotypes. Furthermore, we did not have any robust evidence to believe that the effect of PGS for autism on structural brain phenotypes, either globally or regionally differs by sex. The current results provide no evidence to suggest that sex differences in autism diagnosis and presentation emerge from sex differences in brain structure. However, we note that the sex-stratified GWAS were not particularly well powered, and that the sex-stratified analyses were conducted using smaller sample sizes compared to the unstratified analysis.

Despite the large sample size, our study has a few limitations. First, we focussed only on individuals of genetically inferred European ancestries given both the relatively small number of individuals of other ancestries with neuroimaging and genetic data, and the poor portability of polygenic scores across genetically inferred ancestries [74]. Second, our study does not interrogate how heterogeneity within autism [75] may contribute to differences in results. Third, the current polygenic scores for autism capture only between 1–2% of the total variance for autism [3, 75]. However significant findings from polygenic scores have been corroborated using genetic correlations, which accounts for a larger fraction of the variance in autism likelihood. Finally, the estimates may be biased due to participation bias in both UKB and ABCD [76].

In conclusion, we find robust evidence to suggest that common variants that increase the likelihood for autism are associated with decreased neurite density index. This supports similar findings from smaller-scale neuroimaging, animal and postmortem research. However, this association with neurite density is not specific to autism, and future research needs to investigate whether reduced neurite density is a transdiagnostic marker for neurodevelopmental and mental health conditions.

## Supplementary information

Supplementary Figures

Supplementary Tables

## Data Availability

This study used imaging, genetic and demographic data collected from the UK Biobank Resource under application number 20904, and ABCD. Data are available by request to registered and approved researchers on their corresponding platforms. GWAS summary statistics and cortical morphology data are available through request from the CAM:IDE Data Access Portal (https://portal.ide-cam.org.uk/overview/483) [22]. Prior to the publication of the next iPSYCH PGC GWAS of autism, access to the iPSYCH autism GWAS sumstats [50] can be requested from Jakob Grove and Anders Børglum, while subsequently they will be available for download at https://ipsych.dk/en/research/downloads/.

## Code availability

All analyses were written in R (Version 4.3.1). Mendelian Randomisation was performed using R package TwoSampleMR [77, 78] (https://mrcieu.github.io/TwoSampleMR/) and MRPRESSO [79] (https://github.com/rondolab/MR-PRESSO). The pipelines used to generate processed MRI-derived phenotypes are available through GitHub (UKB, https://github.com/ucam-department-of-psychiatry/UKB; ABCD, https://github.com/ucam-department-of-psychiatry/ABCD. PGS was generated using the code provided by PRScs using “--phi 1e-2”: https://github.com/getian107/PRScs. Genetic correlations were conducted in line with the code provided by the software developers: https://github.com/bulik/ldsc. Details of how glasser regions were mapped onto the networks are provided here: https://github.com/ucam-department-of-psychiatry/maps_and_parcs. All other codes used for this study are available at https://github.com/yg330/autism.img-pgs.
